# Analysis of Template Variations on RNA Synthesis by Respiratory Syncytial Virus Polymerase

**DOI:** 10.3390/v15010047

**Published:** 2022-12-23

**Authors:** Dongdong Cao, Inesh Gooneratne, Cristopher Mera, Jenny Vy, Maurice Royal, Bozun Huang, Yuri Park, Ambika Manjunath, Bo Liang

**Affiliations:** Department of Biochemistry, Emory University School of Medicine, Atlanta, GA 30322, USA

**Keywords:** respiratory syncytial virus (RSV), RNA-dependent RNA polymerase (RdRp), de novo RNA synthesis, back-priming elongation, primer-based elongation, RNA secondary structure, template variations, RNA gel shift assay

## Abstract

Respiratory syncytial virus (RSV) is a significant threat to infants and elderly individuals globally. Currently, there are no effective therapies or treatments for RSV infection because of an insufficient understanding of the RSV viral machinery. In this study, we investigated the effects of the template variations on RNA synthesis by the RSV polymerase through in vitro RNA synthesis assays. We confirmed the previously reported back-priming activity of the RSV polymerase, which is likely due to the secondary structure of the RNA template. We found that the expansion of the hairpin loop size of the RNA template abolishes the RSV polymerase back-priming activity. At the same time, it seemingly does not affect the de novo RNA synthesis activities of the RSV polymerase. Interestingly, our results show that the RSV polymerase also has a new primer-based terminal extension activity that adds nucleotides to the template and primer in a nonspecific manner. We also mapped the impact of the RNA 5′ chemical group on its mobility in a urea-denaturing RNA gel shift assay. Overall, these results enhance our knowledge about the RNA synthesis processes of the RSV polymerase and may guide future therapeutic efforts to develop effective antiviral drugs for RSV treatment.

## 1. Introduction

Viruses are a growing threat to human health and are the cause of numerous human deaths around the world [1]. Among them, respiratory syncytial virus (RSV) is a major cause of severe respiratory tract diseases in infants, the elderly, and immunocompromised worldwide [2,3,4,5]. RSV infects virtually all children before they are two years old. In 2019, RSV was estimated to cause approximately 3.6 million hospital admissions and 101,400 deaths globally in children aged 0–60 months [4]. Meanwhile, within the elderly population in the United States, RSV infections have been attributed to approximately 177,000 hospital admissions and 14,000 deaths annually [5,6].

Unfortunately, no licensed vaccine or effective antiviral therapy is available to prevent or treat RSV infections [4,7,8]. Palivizumab, approved for medical use in 1998, is a humanized monoclonal antibody targeting the fusion protein of RSV, and thus inhibits viral entry into the cell. However, it can only help prevent severe RSV disease in high-risk infants [9]. Another antiviral, Ribavirin, a guanosine analog targeting viral RNA synthesis, is no longer in use due to its limited efficacy and significant safety concerns [10]. The limitations of RSV treatment reveal a need for a better understanding of the viral machinery for developing effective therapeutic treatments against RSV.

As a non-segment, negative-sense (NNS) RNA virus, RSV belongs to the family of *Pneumoviridae* in the order of *Mononegavirales* [11]. Both viral genome replication and viral gene transcription are carried out by the RSV polymerase, similar to other viruses in *Mononegavirales*, such as rabies virus (RABV) and vesicular stomatitis virus (VSV). The RSV polymerase comprises an essential cofactor phosphoprotein (P) and a large (L) protein that catalyzes three distinct enzymatic functions: RNA-dependent RNA polymerase (RdRp), polyribonucleotidyltransferase (PRNTase or Capping), and cap methyltransferase (MTase) [12]. As the key component involved in RSV amplification, the RSV polymerase is an attractive target for antiviral drugs.

Both the RSV genome and antigenome RNAs are encapsidated by the nucleoprotein (N), and each RSV N protein covers seven nucleotides (nts) [13,14,15]. The 44-nt leader (Le) at the 3′ end of the genome and 155-nt trailer complementary (TrC) at the 3′ end of the antigenome serve as promoters for RNA synthesis by the RSV polymerase [16,17]. Both Le and TrC promoters contain two initiation sites, +1 and +3. Genomic and antigenomic RNA replication initiates at position +1 of the Le and TrC promoters. Alternatively, transcription initiates at position +3 of the Le promoter. The +3 initiation at the TrC promoter only generates short (less than 25 nts) transcripts [18,19,20,21,22]. Fearns group demonstrated that the initiating nucleotides are loaded into RdRp independently of the template [23,24]. Our previous work also showed that the minimal length of the RNA template for in vitro de novo RNA synthesis is 8 nts, and the positions 3, 5, and 8 of the promoter sequences are essential for the RNA synthesis activity of the RSV polymerase [25]. In addition to the de novo and primer-based RNA synthesis, the RSV polymerase exhibits a back-priming activity in which the RSV polymerase recognizes a specific secondary structure of the RNA template and adds nucleotides to the 3′ end of the template [22]. In RSV-infected cells, this back-priming activity is only observed in the TrC promoter, not in the Le promoter [26]. However, in vitro assays suggest that both Le and TrC promoters show back-priming activity by the RSV polymerase [22,26]. The back-priming activities are also observed in some other NNS viruses, such as human metapneumovirus (HMPV) [27], Ebola virus [28], and borna disease virus (BDV) [29]. Despite enriching insights from previous studies, the in-depth analysis of the substrate variations on back-priming RNA synthesis activities remains to be illustrated in RSV.

In this study, we confirmed the back-priming activity of the RSV polymerase and that only TrC templates longer than 18 nts show back-priming activity. Additionally, our results indicated that the enlargement of the loop size in the secondary structure of the RNA templates might not influence the de novo RNA synthesis but abolish the back-priming activity due to steric hindrance. We also observed a primer-based terminal extension activity by the RSV polymerase in which the nucleotide added to the primer and template is nonspecific. Together, these results illustrate factors critical to back-priming activity and present a previously unknown primer-based terminal extension activity of RSV polymerase. Thus, our findings may facilitate efforts to develop effective non-nucleotide allosteric or nucleotide analog inhibitors, given that viral polymerases are an attractive target for antiviral drug designs [30]. 

## 2. Materials and Methods

### 2.1. Expression and Purification of the RSV Polymerase (L–P complex)

The expression and purification of the RSV polymerase (L–P complex) have been described previously [31]. The helper plasmids of codon-optimized sequences of the RSV (strain A2) L and P proteins were provided as a generous gift from Martin Moore (Emory University, Atlanta, GA, USA). The L and P genes were subcloned into the pFastBac Dual vector (Invitrogen, Waltham, MA, USA) with the RSV L gene at open reading frame 1 (ORF1) and the RSV P gene at ORF2. A 6×His tag was added to the N terminus of the RSV L protein, separated by a TEV protease cleavage site. Then, the recombinant pFastBac Dual vector was transformed into *Escherichia coli* DH10Bac for bacmid DNA generation. The Cellfectin II reagent (Thermo Fisher Scientific, Waltham, MA, USA) was used to transfect the bacmid DNA into Sf21 cells to obtain the recombinant baculoviruses. Sf21 cells were infected by the recombinant baculoviruses in suspension culture and harvested 72 h post-infection by centrifugation for 15 min at 1000× *g*. Cells were resuspended in lysis buffer (50 mM sodium phosphate [pH 7.4], 300 mM NaCl, 6 mM MgSO_4_, 10% glycerol, 0.2% NP-40, EDTA-free protease inhibitor), lysed with a homogenizer, and clarified through centrifugation for 60 min at 16,000× *g*. The clarified lysate was incubated with Co2+-NTA agarose resin (GoldBio, St Louis, MO, USA) and washed with wash buffer (50 mM sodium phosphate [pH 7.4], 300 mM NaCl, 6 mM MgSO_4_, 10% glycerol, 10 mM imidazole), and the RSV L–P complexes were eluted with elution buffer (50 mM sodium phosphate [pH 7.4], 300 mM NaCl, 6 mM MgSO_4_, 10% glycerol, 250 mM imidazole). The eluted sample was then treated with TEV enzyme and applied to Co^2+^-NTA agarose resin. The flowthrough sample was applied to a heparin column and further purified by size exclusion chromatography with gel filtration buffer (25 mM HEPES [pH 7.4], 300 mM NaCl, 6 mM MgSO_4_, 0.5 mM tris(2-carboxyethyl) phosphine hydrochloride [TCEP]) using a Superose 6 Increase 10/300 GL column (GE Healthcare, Chicago, IL, USA). SDS-PAGE analyzed the quality of purified proteins. The pure proteins were flash-frozen in liquid nitrogen and stored in 30-μL aliquots at −80 °C for further use. 

### 2.2. In Vitro RNA Synthesis Assay

The RNA promoter sequences with different lengths from the trailer complementary (TrC) region of the antigenome, such as TrC14, TrC21, and TrC25, were used in the RNA synthesis assay. All RNA oligonucleotides were chemically synthesized by Integrated DNA Technologies (Coralville, IA, USA) or Horizon Discovery (Waterbeach, UK), which have hydroxyl groups at both 3′ and 5′ terminals. Radioactive isotope-labeled nucleotides [α-^32^P]-GTP, [α-^32^P]-ATP, [α-^32^P]-UTP, and [γ-^32^P]-ATP were purchased from Perkin Elmer (Waltham, MA, USA). The reaction mixtures containing 2 μM RNA template (without or with 2 μM primer), the RSV L–P complexes (∼300 ng RSV L), NTPs (ATP, CTP, and UTP each at 1.25 mM and GTP at 50 μM with 5 μCi of [α-^32^P] GTP) (figure legends indicate the details of NTPs for each reaction mixture), and reaction buffer (50 mM Tris-HCl [pH 7.4], 8 mM MgCl_2_, 5 mM dithiothreitol, 10% glycerol) are in a final volume of 20 μL. The reaction mixtures were incubated at 30 °C for 2 h and heated to 90 °C for 5 min, and then 5 μL of the stop buffer (90% formamide, 20 mM EDTA, 0.02% bromophenol blue) was added to each reaction mixture. Other radioactive isotope-labeled nucleotides ([α-^32^P] ATP, [α-^32^P] UTP, and [γ-^32^P] ATP) were incubated similarly as [α-^32^P] GTP (figure legends indicate the details for each reaction mixture). The isotope-labeled nucleotides with the same concentration were freshly purchased and used for the reactions. For clarity, we directly compared only the reaction mixtures containing the same radioactive isotope-labeled NTPs. The RNA products were analyzed by electrophoresis on a 20% polyacrylamide gel containing 7 M urea in a Tris-borate-EDTA buffer, followed by phosphorimaging with a Typhoon FLA 7000 scanner (GE Healthcare, Chicago, IL, USA). 

### 2.3. RNA Phosphorylation with T4 Polynucleotide Kinase (T4 PNK)

The molecular weight ladders were generated by labeling Tr3, Tr5, Tr7, Tr14, Tr21, and Tr25 with [γ-^32^P] ATP using T4 polynucleotide kinase (M0201L, NEB, Ipswich, MA, USA) following the protocols of the manufacturer (NEB, Ipswich, MA, USA). Briefly, 100 pmol RNA oligonucleotides were incubated with 50 pmol of [γ-^32^P] ATP and 20 units of T4 PNK in 20 μL 1× T4 PNK reaction buffer at 37 °C for 30 min. Then, the 20 μL reaction mixture was heat-inactivated by incubating at 65 °C for 20 min. The radioactive-labeled RNA ladders with 5′ monophosphate group were frozen and stored at −20 °C. 

Some RNA samples from in vitro RNA synthesis assay were treated with T4 PNK to evaluate the influence of the 5′ chemical groups on the RNA migration rates in electrophoresis. Subsequently, 5 μL out of 20 μL in vitro RNA synthesis assay reaction mixture was added by 1 μL ATP (10 mM), 1 μL (10 units) T4 PNK, 1 μL T4 PNK reaction buffer (10×), and 2 μL nuclease-free water. Then, the reaction mixture was incubated at 37 °C for 30 min and then heat-inactivated by incubating at 65 °C for 20 min. The same amount of RNA products before and after T4 PNK treatment were loaded onto the RNA denaturing gel for further analysis.

## 3. Results

### 3.1. Template Back-Priming and the Impact of Hairpin Loop Size on RNA Synthesis Activity of the RSV Polymerase

**Figure 1:**
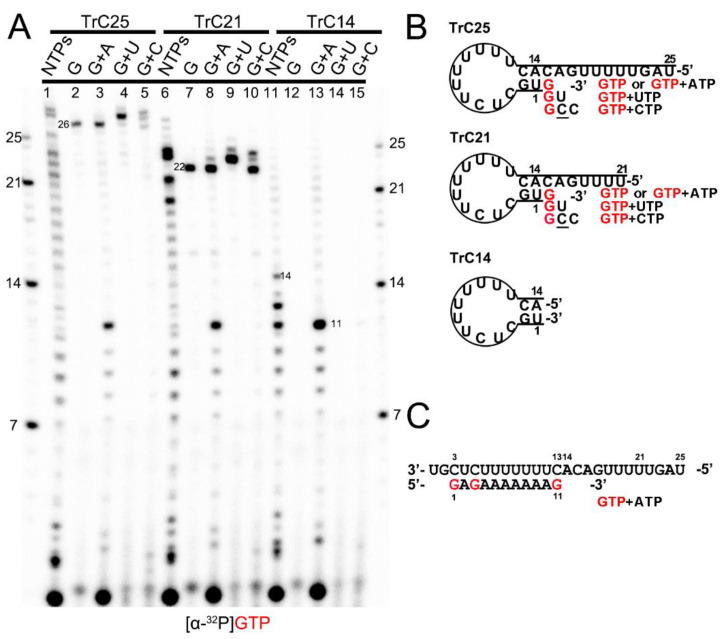
Back-priming-based RNA elongation with different NTPs. (**A**) The RNA synthesis of the templates TrC14, TrC21, and TrC25 in the presence of different NTPs (NTPs, GTP only, GTP+ATP, GTP+UTP, or GTP+CTP) by RSV polymerase (5 μCi of [α-^32^P] GTP was added in each reaction beside other NTPs). (**B**) The back-priming secondary structures of TrC25, TrC21, and TrC14 are predicted by mFold. The CTP in the wobble base pair A-C is underlined. (**C**) RNA synthesis starts at position 3 of the templates TrC25, TrC21, and TrC14 in the presence of GTP and ATP. Red G or GTP indicates radiolabeled GTP.

**Figure 2:**
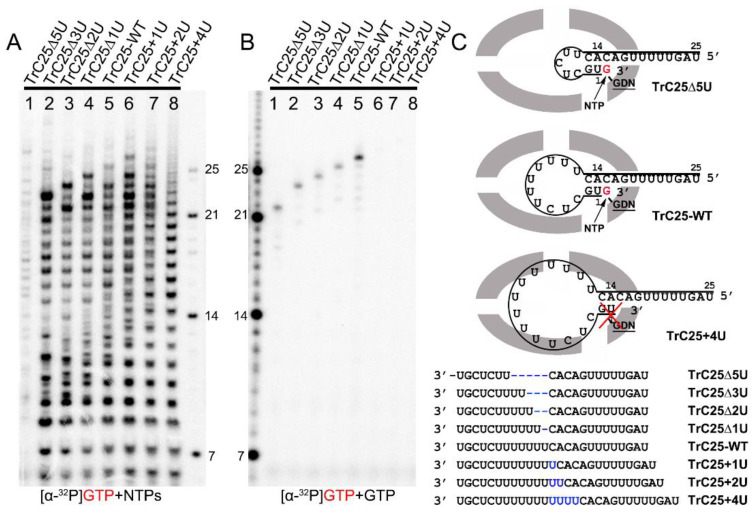
Impact of hairpin loop size on de novo RNA synthesis and back-priming-based 3′ terminal extension. (**A**) De novo RNA synthesis of TrC25-WT and mutants with different numbers of Us at the position 6-12 by the RSV polymerase with NTPs (ATP, CTP, and UTP each at 1.25 mM and GTP at 50 μM with 5 μCi of [α-^32^P] GTP). (**B**) The 3′ terminal extension of wt and mutants of RNA templates TrC25 with GTP only. GTP at 50 μM with 5 μCi of [α-^32^P] GTP was used in the reaction mixtures. (**C**) Sequences and secondary structures of the RSV wt and mutants of TrC25 RNA templates. Residues GDN are the catalytic residues in the RdRp domain that are responsible for RNA polymerization. The red cross indicates no activity towards TrC25+4U. Red G or GTP indicates radiolabeled GTP. The blue Us indicate additional UTP residues that were added to the wt TrC25 template, while the blue dashes indicate Us that were deleted from the wt TrC25 template.

Previous research reported that the 3′ terminal of the RNA template could be extended by the RSV polymerase using a back-priming mechanism [19,22,26,32]. This is because the promoter region of the templates (longer than 14 nts) forms a hairpin loop secondary structure, as predicted by the mFold web server [33] and shown in Figure 1B. As a result, the RSV polymerase can recognize the template as a back-primer and lead to the nucleotide(s) being added to the 3′ end of the template (Figure 1 and Figure 2).

To confirm this mechanism, we tested TrC14, TrC21, and TrC25 (the RNA promoter sequences from the TrC region of the antigenome) with different NTPs (NTPs, GTP only, GTP+ATP, GTP+UTP, and GTP+CTP), and [α-^32^P]-GTP was used to visualize the products (Figure 1A). Our results showed that in the presence of NTPs (ATP, UTP, GTP, and CTP), the RSV polymerase synthesized the RNA products from 1 nt to the length of the templates (Figure 1A, lanes 1, 6, and 11). For TrC21 and TrC25, several bands longer than the templates were detected (Figure 1A, lanes 1 and 6), which may be due to the addition of nucleotides to the templates by back-priming, as they both can form a specific secondary structure (Figure 1B). TrC14 did not produce similar bands larger than 14 nts (Figure 1A, lanes 11) because the secondary structure of TrC14 has a flat end (Figure 1B). In reaction with GTP alone (Figure 1A, lanes 2, 7, and 12), only templates TrC21 and TrC25 had one band that is 1 nt larger than the template (22 and 26 nts) and no band showed up for template TrC14. When GTP and ATP were added (Figure 1, lanes 3, 8, and 13), all of the templates had products from 1 nt to 11 nts (Figure 1A, lanes 3, 8, and 13), which corresponded to the products of +3 de novo synthesis from position 3 to 13 [19,20,21,22,24,34] (Figure 1C). Using GTP and UTP (Figure 1A, lanes 4, 9, and 14), a band 2 nts larger than the templates showed up for templates TrC21 and TrC25. This band corresponded to GU addition to the 3′ end of the templates (Figure 1B). When adding GTP and CTP, three product bands 1, 2, and 3 nts larger than the templates for TrC21 and TrC25 (but not for TrC14) were observed (Figure 1A, lanes 5, 10, and 15). The band 1 nt larger than the template corresponded to a G addition to the 3′ end of the template; the band 2 nts larger than the template possibly corresponded to a GC addition to the 3′ end of the template with a wobble base pair of A-C; the band 3 nts larger than the template possibly corresponded to a GCC addition to the 3′ end of the template with one wobble base pair A-C [35,36,37] (Figure 1B). Due to the wobble base pair A-C, the bands of 2 and 3 nts larger than the templates were weaker (Figure 1A, lanes 5 and 10), suggesting a lower activity. In summary, our results agreed well with the back-priming mechanism of 3′ terminal extension to the template RNAs. 

To investigate the impacts of the hairpin loop variations of TrC25 on the back-priming template elongation and de novo RNA synthesis, we mutated the poly7U at the position 6 to 12 of TrC25 from 7U to 2–10U: 2U (TrC25Δ5U), 4U (TrC25Δ3U), 5U (TrC25Δ2U), 6U (TrC25Δ1U), 8U (TrC25+1U), 9U (TrC25+2U), and 10U (TrC25+4U) (Figure 2). The assay results showed that the de novo RNA synthesis activities of the RSV polymerase towards the templates from TrC25Δ3U to TrC25+1U were similar to each other (Figure 2A, lanes 2–6). In contrast, template TrC25Δ5U generated much fewer products than the others (Figure 2A, lane 1). This difference may be due to the absence of U at position 8, which was previously identified as a key residue of the template [17,25]. Products from templates TrC25+2U and TrC25+4U adopted a different pattern and were predominantly polyadenylated RNA products (Figure 2A, lanes 7–8) due to longer poly U sequences. The U deletions of TrC25 did not influence the back-priming-based RNA elongation (Figure 2B, lanes 1–4), while the U additions of TrC25 abolished the back-priming-based RNA elongation (Figure 2B, lanes 6–8). This suggests that the active pocket of the RSV polymerase does not accommodate back-priming templates with larger loops (Figure 2C). 

### 3.2. The Influence of 5′ Chemical Groups on RNA Mobility in Urea-Denaturing RNA Gel 

This work involved three types of chemical groups at the 5′ end of the RNAs: hydroxyl group, monophosphate group, and triphosphate group. The RNA oligonucleotides ordered from Integrated DNA Technologies or Horizon Discovery contain hydroxyl groups at their 5′ terminals, leading to the RNA products of primer-based or template-based back-priming RNA elongation also having hydroxyl groups at the 5′ terminal. The 5′ chemical group of the RNA ladder is monophosphate, while the RNA products from de novo RNA synthesis have a triphosphate group at the 5′ terminal. 

In Figure 2B, lane 1, we noticed that the length of TrC25Δ5U is 20 nts, and when incubated with GTP only, the product should be 21 nts (5′ hydroxyl group). However, the band shown on the gel (Figure 2B, lane 1) corresponded to 22 nts of the ladder (5′ monophosphate group). We speculated that the 1 nt difference is due to the different migration rates of the RNA’s 5′ chemical groups. To further clarify this, we investigated the influence of the RNA’s 5′ chemical group (hydroxyl group, monophosphate group, and triphosphate group) on its migration rate in RNA denaturing gel. 

**Figure 3:**
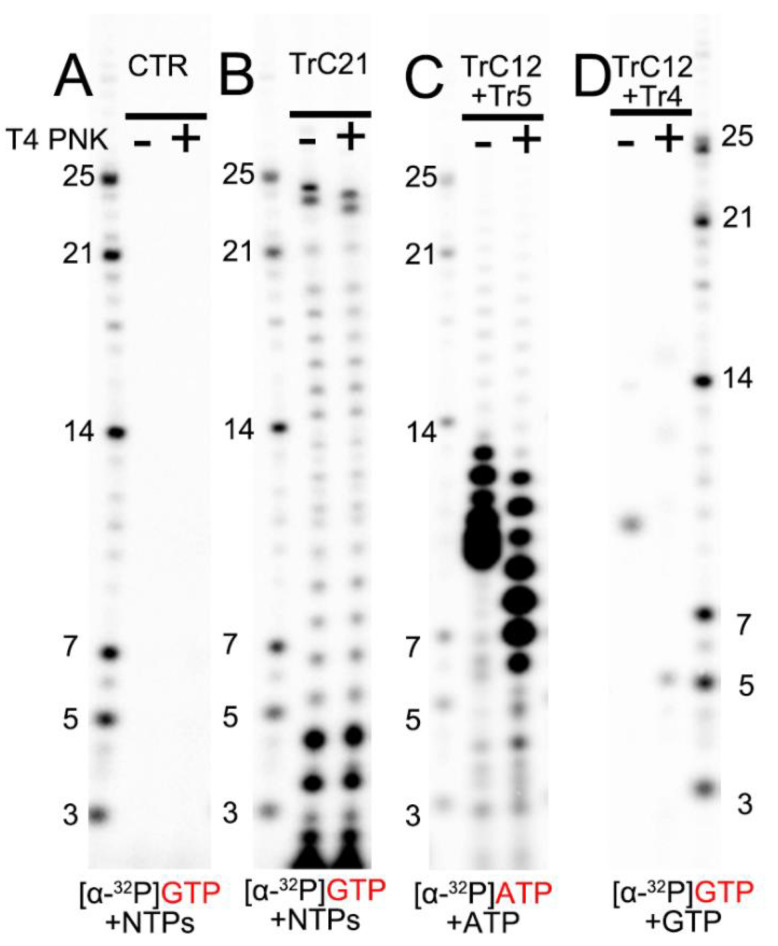
The impact of RNA 5′ end modification on RNA mobility in Urea-denaturing polyacrylamide RNA gel. (**A**) Control: the RSV polymerase + NTPs and [α-^32^P] GTP w/o T4 PNK treatment. (**B**) In vitro RNA synthesis of TrC21 by RSV polymerase in the presence of NTPs + [α-^32^P] GTP with/without T4 PNK treatment. (**C**) In vitro RNA synthesis of the template TrC12 paired with primer Tr5 by RSV polymerase in the presence of NTPs + [α-^32^P] ATP with/without T4 PNK treatment. (**D**) In vitro RNA synthesis of the template TrC12 paired with primer Tr4 by RSV polymerase in the presence of [α-^32^P] GTP with/without T4 PNK treatment. Red GTP and ATP indicate radiolabeled GTP and ATP, respectively.

To compare the migration rates of the de novo RNA synthesis products (5′ triphosphate group), we loaded RNA ladders (5′ monophosphate group), templated-based or primer-based elongation products (5′ hydroxyl group), RNA products from the assays before (−) and after (+) T4 PNK treatment (transfer 5′ hydroxyl RNA to 5′ monophosphate RNA) onto the RNA denaturing gels (Figure 3). The de novo RNA synthesis of the TrC21 template by the RSV polymerase showed that 5′ triphosphate group RNA products from de novo RNA synthesis matched well with the RNA ladders containing a 5′ monophosphate group (Figure 3B, bands ≤ 21 nts). T4 PNK treatment did not impact the migrations of de novo RNA synthesis products with a 5′ triphosphate group. However, the template-based elongation products (5′ hydroxyl group), such as the top 2 bands of the products from the TrC21 template (Figure 3B), migrated about 1 nt slower than that with T4 PNK treatment (5′ monophosphate group). To test short RNAs, we treated the products from template TrC12 and primer Tr5 with ATP only, which range from 6–12 nts, with T4 PNK. Before T4 PNK treatment, the products were located at the 9–13 nts region of the RNA ladders, and some small products were migrated together (Figure 3C). With T4 PNK treatment (5′ hydroxyl groups were transferred to 5′ monophosphate groups), the products shared the same 5′ chemical group with the RNA ladders and matched well with the RNA ladder positions indicative of 6–12 nts (Figure 3C). The product from template TrC12 and primer Tr4 with GTP only was Tr5 at the length of 5 nts. However, it migrated to the location between 9 and 10 nts of the RNA ladder without T4 PNK treatment, and once treated with T4 PNK, it migrated to the location corresponding to the RNA ladder of 5 nts (Figure 3D). Thus, there was about 5 nts difference between 5′ hydroxyl and 5′ monophosphate RNAs with a length of 5 nts. Taken together, 5′ triphosphate RNA migrated similarly to 5′ monophosphate RNA, while 5′ hydroxyl RNA migrated about 1 nt slower than 5′ monophosphate RNA when the RNA length is 12–24 nts and 1–5 nts slower when the RNA length is 5–12 nts.

### 3.3. The Effects of Primers on the Terminal Extension Activity by RSV Polymerase

**Figure 4:**
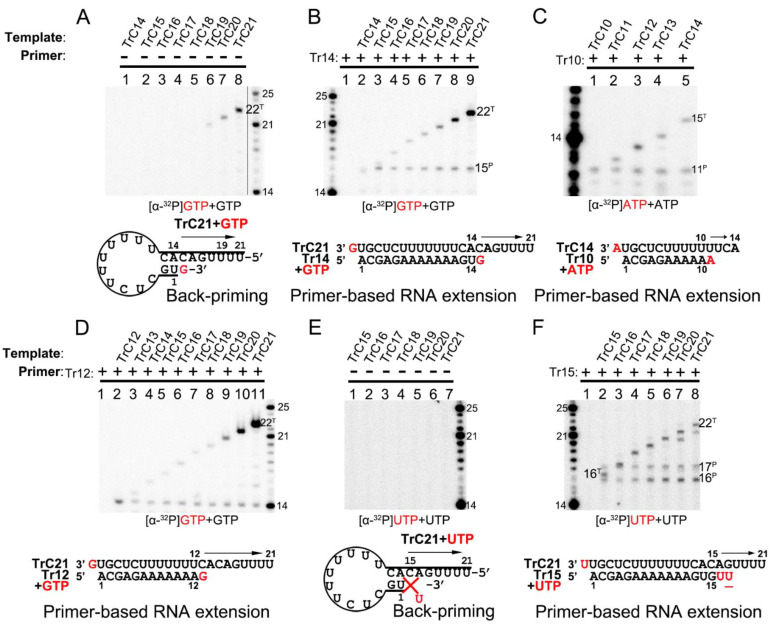
Terminal nucleotide(s) addition to the templates and primers by RSV polymerase. (**A**) Back-primer secondary-structure-based 3′ terminal extension of the templates from TrC14 to TrC21 using GTP only. GTP at 50 μM with 5 μCi of [α-32P] GTP was used in the reaction mixtures. (**B**) Primer-based terminal extension of the templates from TrC14 to TrC21 with primer Tr14 and GTP only. GTP at 50 μM with 5 μCi of [α-32P] GTP was used in the reaction mixtures. (**C**) Primer-based terminal extension of templates from TrC10 to TrC14 with primer Tr10 in the presence of ATP. ATP at 50 μM with 5 μCi of [α-32P] ATP was used in the reaction mixtures. (**D**) Primer-based terminal extension of templates from TrC12 to TrC21 with primer Tr12 in the presence of GTP. GTP at 50 μM with 5 μCi of [α-32P] GTP was used in the reaction mixtures. The 5′ hydroxyl RNA (RNA products extended from template or primer) migrates about 1 nt slower than 5′ monophosphate RNA (RNA ladders) when the RNA length is 12–24 nts (See Section 3.2 in Results), leading to the product from primer Tr12 at the length of 13 nts close to the ladder 14 nts. (**E**) Back-primer secondary-structure-based 3′ terminal extension of the templates from TrC15 to TrC21 using UTP only. UTP at 50 μM with 5 μCi of [α-32P] UTP was used in the reaction mixtures. Since U can not pair with C, no products were detected. (**F**) Primer-based terminal extension of the templates from TrC15 to TrC21 with primer Tr15 and UTP only. UTP at 50 μM with 5 μCi of [α-32P] UTP was used in the reaction mixtures. The UTP in the wobble base pair G-U is underlined. The superscript labels indicate that the band is generated from primer (^P^) or template (^T^). The red G or GTP, A or ATP, and U or UTP indicate radiolabeled GTP, ATP, and UTP, respectively.

The RNA secondary structure predicted by mFold web server indicated that TrC promotor sequences longer than 14 nts could form a back-priming secondary hairpin loop structure, allowing RNA elongation at the 3′ end of the template by the RSV polymerase. However, the assay results of RNA elongation on the templates TrC14 to TrC21 (Figure 4A) showed that not all templates longer than 14 nts had back-priming-based elongation products; only longer templates, such as TrC19 to TrC21, could generate a product 1 nt longer than the templates with GTP only (Figure 4A, lanes 6–8). In addition, the density of the bands gradually increased along with the increase in the template length, indicating a higher RNA elongation activity towards longer templates (Figure 4A, lanes 6–8).

Interestingly, adding primer Tr14 to the templates TrC14 to TrC21 generated two products by the RSV polymerase, with one product 1 nt longer than the primer and another product 1 nt longer than the template (Figure 4B, lanes 2–9) with GTP only. The primer Tr14 alone generated no product by RSV polymerase (Figure 4B, lane 1). The product 1 nt longer than the primer (15 nts) was the primer-based RNA elongation, and the density of this band was consistent (Figure 4B). However, the products 1 nt longer than the templates applied to all the templates from TrC14 to TrC21 and had increased intensity along with the increase in the template length, suggesting that the addition of the primers promoted the terminal extension of the template (Figure 4B). The nucleotide addition to the templates from TrC19 to TrC21 may also come from the back-priming activity of RSV polymerase.

To determine whether the addition to the templates is back-priming-based, we also evaluated some shorter templates, from TrC10 to TrC14, in which TrC10 to TrC13 did not form back-priming secondary structures (Figure 4C). In our results, the template TrC10 with primer Tr10 produced a band of 11 nts when incubated with ATP only (Figure 4C, lane 1). From TrC11 to TrC14, they all generated two bands in the presence of primer Tr10 and ATP (Figure 4C, lanes 2–5) by RSV polymerase: one band at the length of 11 nts, which was 1 nt added to the primer by primer-based RNA elongation; another band 1 nt longer than the length of the template, which was not from back-priming activity of RSV polymerase as the templates are too short to form the back-priming secondary structures, suggesting a new primer-based terminal extension to the templates (Figure 4C). Comparable results were observed in the assay of the templates from TrC12 to TrC21 with primer Tr12 in the presence of GTP (Figure 4D). Two clear bands were visible on the gel: one band at the length of 13 nts represented the product of one GTP being added to the primer Tr12 (the product from primer Tr12 at the length of 13 nts was close to the ladder 14 nts due to that the 5′ hydroxyl RNA (RNA products extended from template or primer) migrated about 1 nt slower than 5′ monophosphate RNA (RNA ladders) when the RNA length was 12–24 nts (See Section 3.2 in Results)). Another band 1 nt longer than the template represented one GTP added to the templates from TrC12 to TrC21 (Figure 4D, lanes 2–11). This result further confirmed that the addition to the template, in this case, was not back-priming-based, as TrC12 and TrC13 cannot form the secondary structures required for back-priming.

Furthermore, we tested the templates TrC15 to TrC21 with radioactively labeled UTP in the presence and absence of the primer Tr15 (UTP is the NTP adding to primer-based RNA elongation for primer Tr15 but not to the back-priming-based RNA elongation which should be GTP) (Figure 4E,F). Without primer Tr15, we observed no product around 15 nts for all templates ranging from TrC15 to TrC21 (Figure 4E). Once the primer Tr15 was added to the reaction mixtures, multiple bands were shown on the gel (Figure 4F). All samples except control (lane one, the RSV polymerase only) shared two bands of 16 and 17 nts: the 16-nt band represented one UTP being added to the primer Tr15 using TrC15 to TrC21 as templates; the 17-nt band may represent two UTPs being added to the primer Tr15 with one wobble base pair of G-U (Figure 4F, lanes 2 to 8). Another band 1 nt longer than the template was one UTP added to the template. This indicated that with a primer Tr15, UTP could be added to the templates TrC15 to TrC21 by the RSV polymerase (Figure 4F). 

Interestingly, both GTP and UTP could be added to the templates TrC15 to TrC21 by the RSV polymerase in the presence of a primer, as shown in Figure 4B,D,F. From the results shown in Figure 4C,D, both ATP and GTP could be added to the templates TrC12 to TrC13 by the RSV polymerase in the presence of a primer. These results together suggested that the NTP added to the template is nonspecific.

### 3.4. Primer-Based Terminal Extension to the Template by the RSV Polymerase Is NTP-Nonspecific

**Figure 5:**
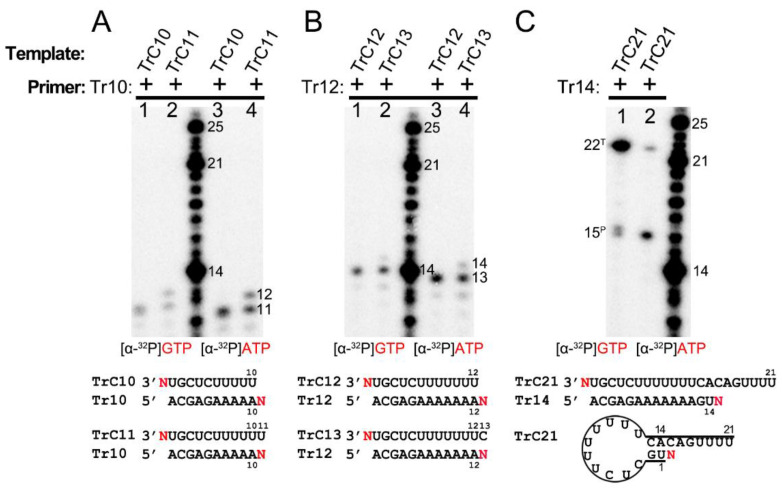
Primer-based terminal extension activity of RSV polymerase is NTP nonspecific. (**A**) Primer-based RNA terminal extension of template TrC10 and TrC11 with primer Tr10 in the presence of [α-^32^P] GTP or [α-^32^P] ATP. (**B**) Primer-based RNA terminal extension of template TrC12 and TrC13 with primer Tr12 in the presence of [α-^32^P] GTP or [α-^32^P] ATP. (**C**) Primer-based RNA terminal extension of template TrC21 with primer Tr14 in the presence of [α-^32^P] GTP or [α-^32^P] ATP. The superscript labels indicate the band is generated from primer (^P^) or template (^T^). ATP at 50 μM with 5 μCi of [α-^32^P] ATP and GTP at 50 μM with 5 μCi of [α-^32^P] GTP were used in the reaction mixtures as indicated. The products from [α-^32^P] GTP are slightly larger than that from [α-^32^P] ATP. Red N represents [α-^32^P] ATP or [α-^32^P] GTP. The red GTP and ATP indicate radiolabeled GTP and ATP, respectively.

To figure out the NTP specificity of the terminal extension, we used two different radioactively labeled NTPs, [α-^32^P]-GTP and [α-^32^P]-ATP, to test templates and corresponding primers at different lengths (Figure 5). The templates TrC10 and TrC11 were incubated with primer Tr10 in the presence of [α-^32^P] GTP (Figure 5A, lanes 1 and 2) or [α-^32^P] ATP (Figure 5A, lanes 3 and 4). One 11-nt product was observed upon using template TrC10 and primer Tr10 for both ATP and GTP, which may be the 1-nt product being added to the template or primer (Figure 5A, lanes 1 and 3). Additionally, the RSV polymerase produced products at the length of 11 and 12 nts with template TrC11 and primer Tr10, which may correspond to 1 nt being added to the template and primer, respectively (Figure 5A, lanes 2 and 4). The products with GTP were slightly larger than those with ATP due to the different molar masses of GTP (523.18 g/mol) and ATP (507.18 g/mol) (Figure 5A). Templates TrC12 and TrC13 with primer Tr12 were also tested with [α-^32^P] ATP and [α-^32^P] GTP, and similar results were observed: one band of 13 nts for TrC12+Tr12; two bands of 13 and 14 nts for TrC13+Tr12. With respect to the TrC13+Tr12 condition, the 14-nt band was weaker than that of 13-nt (Figure 5B), indicating a lower activity for template terminal addition than primer terminal extension. The products with GTP were also slightly larger than those with ATP (Figure 5B). With template TrC21 and primer Tr14, two bands of 15 and 22 nts were observed in the presence of both ATP and GTP (Figure 5C). Interestingly, with ATP, the band of 15 nts (primer nucleotide addition) was more enriched than the band of 22 nts (template nucleotide addition). However, with GTP, the result was reversed (Figure 5C). This may be because TrC21 could form a specific secondary structure required for back-primer-based elongation, and GTP is the incoming NTP. Overall, our results showed that the RSV polymerase could add either ATP or GTP to the same template and primer, suggesting a new primer-based terminal extension activity by RSV polymerase in which the addition of NTP is nonspecific.

## 4. Discussion

### 4.1. The Back-Priming Mechanism of the RSV Polymerase 

**Figure 6:**
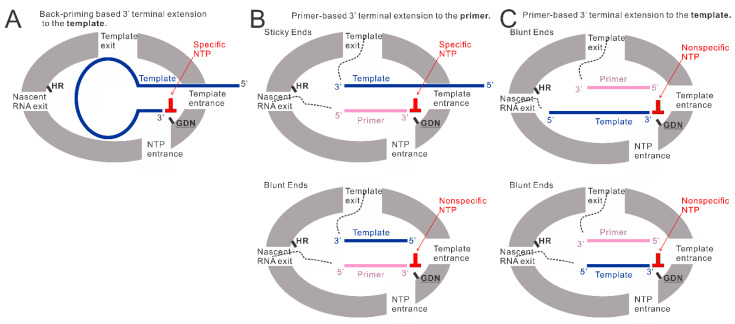
The models of back-priming-based and primer-based 3′ terminal extension to the template and primer. (**A**) The model of back-priming-based 3′ terminal extension to the template by RSV polymerase. It may incorporate a wobble base pair with low activity only when only a high concentration of nonspecific NTP was added to the reaction mixture. (**B**) The model of primer-based 3′ terminal extension to the primers. The incoming NTP will be specifically added to the sticky end when the template is longer than the primer. When the supplemented NTP is nonspecific, a wobble base pair may be introduced into the product by RSV polymerase with low activity. While the template is the same length as the primer, the RSV polymerase may add a nonspecific NTP to the blunt end of the double-strand RNA at the 3′ end of the primer. (**C**) The model of primer-based 3′ terminal extension to the templates. The double-strand RNA (template paired with the primer) may enter the active pocket to access the GDN catalyze motif in RdRp domain, allowing a nonspecific NTP addition to the 3′ blunt end of the template. GDN are the catalytic residues in the RdRp domain responsible for RNA polymerization. HR are the catalytic residues in the Cap domain responsible for mRNA capping.

Our previous research observed that the RSV polymerase produces premature RNAs in vitro with naked RNA templates from the Le or TrC region [25]. Additionally, when TrC25 was used as the template, two bands larger than 25 nts were detected on the RNA denaturing gel. This may be due to the back-priming activity of the RSV polymerase on the RSV promoter templates [19,22,26]. Our results here confirmed the back-priming activity of RSV polymerase, which adds G, GU, or GCC (underlined C indicates CTP in the wobble base pair A-C) to long RNA promoter sequences from the TrC region of the antigenome (TrC21 and TrC25), but not short one (TrC14) (Figure 1). Specifically, in vitro assay testing with different lengths of TrC promoter templates from TrC14 to TrC21 showed that the back-priming activity of RSV polymerase requires the templates to be long enough (≥19 nts) for 3′ terminal extension (Figure 4A), which suggests some restrictions on the back-priming-based 3′ extension activity. One possibility is that the overhang sequence after position 14 of the back-priming secondary structure should be long enough to interact with the residues in the template entrance channel to achieve a high affinity or stable conformation for further RNA synthesis (Figure 6A).

We also showed that the additions to the template (TrC21 and TrC25) might be limited to 3 nts, and further RNA elongation by back-priming was rarely detected compared with the first 3 nts addition (Figure 1), which may be due to the hairpin loop blocking the exit of the template from the active pocket and preventing further elongation of the nascent RNA product (Figure 6A). Further investigation of the hairpin loop showed that the deletions of the loop size did not influence the back-priming activity toward the templates. In contrast, the increase in the loop size yielded no product from back-priming RNA elongation (Figure 2B), indicating the tolerance of the active pocket of the polymerase toward the substrate (Figure 6A). 

### 4.2. The Primer-Based Terminal Extension Activity by the RSV Polymerase 

The back-priming-based RNA elongation by RSV polymerase required a sufficiently long (≥19 nts) specific template. Surprisingly, when a corresponding primer, such as Tr10, Tr12, Tr14, and Tr15, was added to the reaction, both the template (from TrC10 to TrC21) and primer showed terminal extension activity by RSV polymerase when incubated with a corresponding singular NTP (Figure 4 and Figure 5). The nucleotide addition to the primer is primer-based RNA elongation when the template is longer than the primer (Figure 6B, top model), which may incorporate a wobble base pair with low activity when only a nonspecific high concentration NTP is added to the reaction mixture (Figure 1 and Figure 4F). We also observed that when the template was the same length as the primer, both the template and the primer were added by an NTP in the presence of the RSV polymerase (Figure 4B,D,F, lane 2; Figure 4C, lane 1; Figure 5A,B, lanes 1 and 3), indicating that the RSV polymerase can add a nucleotide to a blunt end double-strand RNA (Figure 6B, bottom model, and Figure 6C). The mechanism of primer-based terminal extension to the template is unclear. We hypothesize that when the template is paired with the primer, the blunt end at the 3′ terminal of the template can be extended by one nucleotide by RSV polymerase (Figure 6C).

Furthermore, templates from TrC15 to TrC20 could be added by either GTP (Figure 4B,D) or UTP (Figure 4F), template TrC21 could be added by GTP (Figure 4A,B,D), UTP (Figure 4F), or ATP (Figure 5C), and templates TrC12 and TrC13 could be added by either ATP (Figure 4C and Figure 5B) or GTP (Figure 4D and Figure 5B). These results suggested the primer-based 3′ nucleotide addition to the template is NTP nonspecific. We hypothesize that the blunt end (3′ end of the template) of the short double-strand RNA (template-primer) may enter the active pocket of RSV polymerase to access the catalyze motif of “GDN” at the RdRp domain allowing a nonspecific nucleotide addition to the template (Figure 6C). 

The biological roles of back-priming and primer-based terminal extension activities in RSV replication remain largely unclear. Previous research identified that back-priming-based 3′ terminal extension was only detected in TrC promoter of antigenome, not Le promoter of the genome in RSV-infected cells [22]. However, the in vitro assay showed that back-priming occurred in both TrC and Le promoters, and TrC promoter has a higher back-priming activity than that of Le promoter [22,26]. The in vitro RNA synthesis assay with the naked TrC templates showed that the back-priming activity also inhibits antigenome promoter activity [22]. It was thought that the back-priming activity at the TrC promoter of antigenome might involve the negative regulation of the replication from antigenome RNA to genome RNA, especially when the N protein is not enough to encapsidate the full-length antigenome RNA [22,26]. In addition, given that the length of genomic RNA cannot be divisible by seven (one N monomer covers 7 nts [13,14,15]), the formation of the back-priming secondary structure of the TrC promoter might protect the unencapsidated terminal RNA of antigenome from degradation by cellular exonucleases [22]. We identified the primer-based terminal extension activity using naked RNA templates and primers, which might apply to RSV-infected cells. This activity might also involve the negative regulation of the TrC promoter and protect TrC terminal from cellular exonuclease as it adds random NTP to the 3′ terminal of the templates. Further research is needed to clarify the mechanisms and structures of the back-priming activity and primer-based terminal extension activity of RSV polymerase.

### 4.3. Implications for the Development of RSV Antiviral Drugs 

Currently, there is a considerable effort to develop an antiviral drug to treat RSV infections [38,39]. Generally, in such endeavors, the most successful drugs have targeted enzymes that are essential to the replication process of the virus [30]. Moreover, the L protein of RSV, which carries out an RdRp function, is an attractive target for therapeutic intervention [40]. Two main classes of polymerase inhibitors, non-nucleotide allosteric inhibitors, and nucleotide analog inhibitors, were investigated for the RSV polymerase. The former works by binding to a site on the polymerase that is not the active site and impeding polymerase functionality by creating conformational changes to the RdRp or reducing the substrate binding affinity [30]. The nucleotide analog inhibitors, which are sugar or base component alterations containing derivatives of pyrimidines and purines, work by impairing nucleotide chain growth during RNA synthesis of the virus [30]. At present, PC786, which is a non-nucleoside RSV L protein polymerase inhibitor [39,41], and EIDD-2749, a ribonucleoside analog, are examples of some promising new antiviral drugs [42]. 

The results of this study can improve the antiviral drug design targeting the RSV polymerase by providing vital information regarding the RNA synthesis process of RSV polymerase. Specifically, the incorporation of wobble base pairs by the RSV polymerase and the nonspecific NTP addition in primer-based 3′ terminal extension suggests that the RSV polymerase is a low-fidelity polymerase leading to a high mutation rate, indicating the potential development of nucleotide analog antiviral drugs which either block further RNA synthesis or influence the protein translation. The back-priming activity allows RSV polymerase to recognize a specific hairpin loop secondary structure RNA, which may provide a new strategy for RSV antiviral drugs development: design a specific hairpin loop secondary structure RNA that occupies the active pocket of RSV polymerase and prevents its binding to viral genome or antigenome. The impact of the loop size on the RSV back-priming RNA synthesis activity indicates one promising direction for strategy optimization. 

## Data Availability

Not applicable.
